# Predicting carbapenem-resistant *Enterobacteriaceae* infections in pediatric liver transplant recipients

**DOI:** 10.1007/s12519-025-00973-9

**Published:** 2025-09-09

**Authors:** Yang-Yang Wang, Wei-Li Wang, Yan Sun, Wei Zhang, Yun-Tao Zhang, Shun-Liang Gao, Jian Wu, Yan Shen, Zhe-Cheng Zhu, Xue-Li Bai, Qi Zhang, Ting-Bo Liang

**Affiliations:** 1 Department of Hepatobiliary and Pancreatic Surgery, the First Affiliated Hospital, Zhejiang University School of Medicine, No. 79 Qingchun Road, Hangzhou 310003, China; 2 Department of Surgical Intensive Care Unit, Tianjin First Central Hospital, Tianjin, China; 3 Key Laboratory of Organ Transplantation, Tianjin, China; 4 Zhejiang Clinical Research Center of Hepatobiliary and Pancreatic Diseases, Hangzhou, China

**Keywords:** Carbapenem-resistant *Enterobacteriaceae* infection, Pediatric liver transplantation, Prediction, Risk factors

## Abstract

**Background:**

Carbapenem-resistant *Enterobacteriaceae* (CRE) infections can pose a significant risk following pediatric liver transplantations. This study aimed to identify risk factors for CRE infections and develop prediction models for pediatric recipients.

**Methods:**

This study enrolled pediatric patients who underwent liver transplantation between 2017 and 2023. Risk factors for CRE infection were identified using logistic regression analysis. Prediction models were constructed using a training cohort and validated using internal and external validation cohorts. Predictive performance was assessed using receiver operating characteristic curves and area under the curve (AUC).

**Results:**

CRE intestinal colonization before liver transplantation, bile or intestinal leakage and respiratory ribonucleic acid virus infections were independent risk factors for CRE infection after pediatric liver transplantation. Our prediction model comprising all three factors achieved AUC values of 0.724 and 0.738 in the training and internal validation cohorts, respectively. The AUC of an additional model constructed using CRE intestinal colonization and bile or intestinal leakage achieved 0.738 and 0.828 in the internal and external validation cohorts, respectively. Two nomograms were constructed.

**Conclusions:**

Both nomograms accurately predicted CRE infection after liver transplantation. They can facilitate the adoption of essential protective measures in pediatric liver transplant recipients.

**Graphical Abstract:**

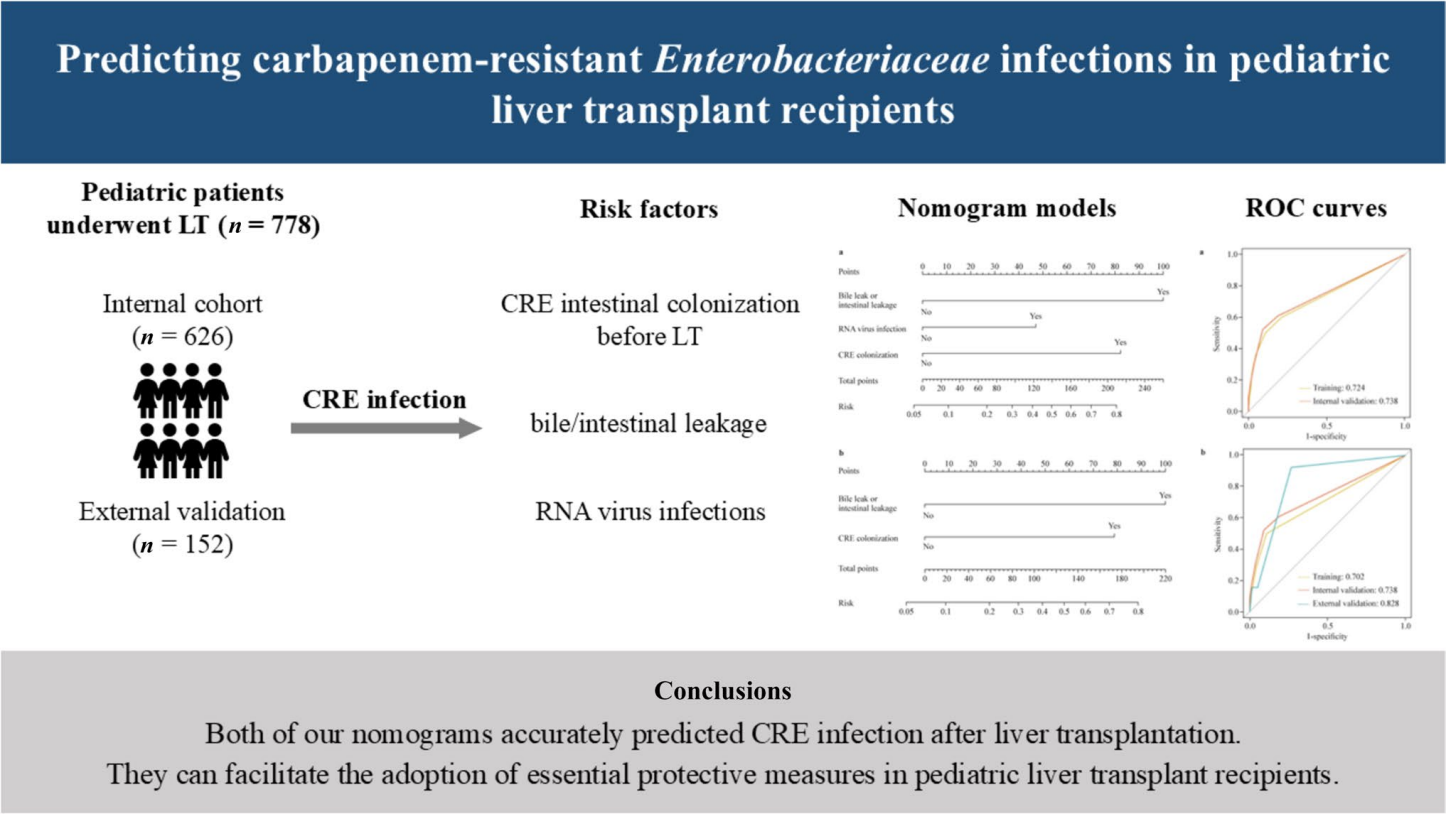

## Introduction

Carbapenem-resistant *Enterobacteriaceae* (CRE) infections have emerged as a global threat in recent years and are associated with high mortality, limited treatments and significant healthcare costs [1, 2]. Patients who have undergone solid organ transplantation are susceptible to CRE infections [3]. Complications following liver transplantation make those patients more susceptible to developing bacterial infections than those who have undergone other organ transplantation [4].

Previous studies have identified a number of risk factors associated with CRE infection in liver transplant patients. These include CRE colonization, acute renal injury, surgical reintervention, rejection and prolonged mechanical ventilation [5–7]. Indeed, Giannella et al. described a predictive model for CRE infections with an acceptable efficacy [5]. Collectively, the above findings have aided the formulation prevention strategies for CRE. These include the implementation of strict infection control [8]. However, these studies primarily focused on adult liver transplantation and lacked pediatric applicability.

Pediatric liver transplant recipients often have diverse underlying conditions and may present with a history of prior surgical procedures, such as Kasai portoenterostomy [9]. In addition, the developing pediatric immune system lacks maturity creating dosing challenges for a range of medications in transplant recipients [10]. In this context the epidemiology of bacterial infections and resistance to treatment differ in pediatric compared to adult populations [11]. Whilst the past decades have witnessed significant advances in pediatric liver transplantation, there is an increasing need to focus on the occurrence of severe CRE infections post transplantation. Moreover, whilst a previous study has described the causative pathogens for bloodstream and intra-abdominal infections [12], data on CRE are limited. In particular the impact of CRE infections on pediatric liver transplantation remains unclear.

The present multicentre analysis aimed to construct a prediction model for CRE infections after liver transplantation in pediatric recipients. The model will help identify pediatric recipients with a high risk of postoperative CRE infection, thereby aiding development of clinically relevant preventive strategies.

## Methods

### Study design

The present study complied with the Declaration of Helsinki and was approved by the Ethics Committee of the First Affiliated Hospital, Zhejiang University School of Medicine (FAHZU, IIT20240071A). All organs were donated voluntarily with written informed consent. Medical records of pediatric patients (< 12 years) who underwent liver transplantation between January 2020 and June 2023 in FAHZU (center 1) and those of patients who presented to Tianjin First Central Hospital (center 2) between August 2017 and August 2018 were reviewed retrospectively. Pediatric patients without rectal microbiological cultures before transplantation were excluded. Data for each recipient was included only once, and for patients with two transplantations in the study period, the data from the second transplantation was analyzed.

Baseline data included age (months), weight, gender, primary disease (transplant indications), pediatric end-stage liver disease scores, pre-transplant CRE colonization, pre-transplant intensive care unit (ICU) stay and operative parameters. In addition, post-transplant complications, CRE infection status and clinical outcomes were recorded. Respiratory ribonucleic acid (RNA) virus infection data in transplant recipients was also collected. Respiratory RNA virus infections that occurred before CRE infections were confirmed through nasopharyngeal swabs or secretions from tracheal intubation using polymerase chain reaction (PCR) or high-throughput (next generation) sequencing. The viral spectrum identified by PCR comprised influenza A and B, parainfluenza (I–III), respiratory syncytial viruses and severe acute respiratory syndrome coronavirus.

### Definition of CRE colonization and CRE infection

According to guidelines provided by the Centers for Disease Control and Prevention, CRE are identified as those *Enterobacteriaceae* non-susceptible to imipenem, meropenem, doripenem or ertapenem, or those strains that have been documented to possess a carbapenemase [13]. Rectal swabs were used to detect intestinal CRE colonization before liver transplantation. CRE colonization was identified according to the guidelines of the 31st edition of the Clinical and Laboratory Standards Institute *Performance Standards for Antimicrobial Susceptibility Testing*, M100 [14]. Specifically, a minimum inhibitory concentration of imipenem, meropenem or doripenem ≥ 4 mg/L or ertapenem ≥ 2 mg/L was used to confirm CRE. Laboratory detection of CRE along with the appearance of infection symptoms were confirmation of infection. Infections that occurred within 90 days post-transplant were identified as postoperative CRE infections.

### Definition of acute graft rejection

Elevated serum transaminase indicated the possibility of acute rejection. Subsequently, experienced pathologists confirmed acute rejection based on the Banff rejection activity index (RAI) [15]; an RAI ≥ 3 identified acute rejection.

### Statistical analysis

Categorical variables were presented using frequencies and percentages and were compared using the Chi-squared or Fisher’s exact tests. Continuous variables were summarized as means or medians as appropriate. Mann–Whitney *U* test was used to compare continuous variables. Logistic regression analyses were utilized to identify independent risk factors associated with CRE infections after liver transplantation. Factors with *P* values < 0.01 chosen after univariate analysis were included in subsequent multivariate analysis. Nomograms were constructed based on identified independent factors. Performance of the prediction models were evaluated using receiver operating characteristic curves (ROC) and area under the ROC curve (AUC). All statistical analyses were performed using R 4.1.2, SPSS v26.0 (IBM, Armonk, NY) and Prism 8.0 (GraphPad Soft Inc., San Diego, CA) software programs. A two-sided *P* value < 0.05 was considered statistically significant.

## Results

### Demographic and baseline patient characteristics

**Fig. 1:**
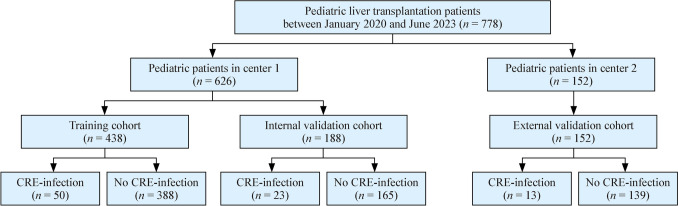
Pediatric liver transplantation study flowchart. *CRE* Carbapenem-resistant *Enterobacteriaceae*

Data from 778 pediatric patients who underwent liver transplantation between August 2017 and June 2023 was included in this study. Six hundred and twenty-six pediatric patients who presented to FAHZU during the study period were randomly divided into training and internal validation cohorts at a ratio of 7:3, adhering to established machine learning conventions and domain-specific standards (*n* = 438 and 188, respectively; Fig. 1). This sample size satisfies the 10 events per variable principle, ensuring adequate statistical power for model development [16]. In addition, 152 pediatric patients from the Tianjin First Central Hospital were included as an external validation cohort. More pediatric patients were female and had CRE intestinal colonization in the external validation cohort than those in the training cohort (*P* = 0.001 and *P* < 0.001, Table 1). Patients in the external validation cohort exhibited higher PLED scores (*P* = 0.006). In the training cohort, fifty (11.4%) patients presented with CRE infections after transplantation; the remaining 388 (88.6%) patients were not infected. The number of patients with CRE infections was 23 (12.2%) and 13 (8.6%) in the internal and external validation cohorts, respectively (*P* = 0.524), demonstrating comparability between cohorts. The majority of baseline characteristics in patients with CRE infections were similar to those not infected (Table 2). However, bile or intestinal leakage, respiratory RNA virus infection and CRE intestinal colonization were observed more frequently in pediatric recipients with CRE infections than those not infected (*P* < 0.001, *P* = 0.005, and *P* < 0.001, respectively). Cefoperazone-sulbactam serves as the first-line agent for perioperative antibiotic prophylaxis, with meropenem used rarely where cefoperazone-sulbactam allergy is confirmed. This selection aligns with established clinical recommendations from prior evidence-based studies [17, 18].

**Table 1:** Patient characteristics in the training, internal validation and external validation cohorts

Variables	Training (*n* = 438)	Internal validation (*n* = 188)	External validation (*n* = 152)	*P*
Age (mon), median (IQR)	7.4 (3.3–145.2)	6.6 (3.5–143.0)	7.0 (4.0–140.0)	0.103
Weight (kg), median (IQR)	7.1 (3.5–40.0)	7.0 (4.5–42.5)	6.5 (4.5–55.0)	0.217
Male, *n* (%)	261 (59.6)	98 (52.1)	65 (42.8)	0.001
Biliary atresia, *n* (%)	371 (84.7)	161 (85.6)	138 (90.8)	0.170
Re-transplantation, *n* (%)	17 (3.9)	3 (1.6)	2 (1.3)	0.131
PELD scores, mean ± SD	13 ± 11	13 ± 10	16 ± 11	0.006
Kasai before LT, *n* (%)	258 (58.9)	104 (55.3)	73 (48.0)	0.066
ICU stay before LT, *n* (%)	33 (7.5)	15 (8.0)	7 (4.6)	0.410
Living donors, *n* (%)	304 (70.0)	128 (68.1)	111 (73.0)	0.611
Operative time (min), mean ± SD	363.7 ± 64.3	362.0 ± 69.8	478.0 ± 83.6	< 0.001
RBC transfusion (U/kg)	0.38 (0–4.29)	0.42 (0–1.80)	0.33 (0–1.14)	0.006
Choledochojejunostomy, *n* (%)	418 (95.4)	181 (96.3)	147 (96.7)	0.755
Bile or intestinal leakage, *n* (%)	30 (6.8)	11 (5.9)	9 (5.9)	0.861
CRE rectal colonization, *n* (%)	45 (10.3)	18 (9.6)	44 (28.9)	< 0.001
CRE infection, *n* (%)	50 (11.4)	23 (12.2)	13 (8.6)	0.524

*PELD* pediatric end-stage liver disease, *LT* liver transplantation, *ICU* intensive care unit, *RBC* red blood cell, *CRE* carbapenem-resistant *Enterobacteriaceae*, *IQR* interquartile range, *SD* standard deviation

**Table 2:** Demographic and baseline characteristics of patients in the training cohort

Variables	CRE infection (*n* = 50)	Non-CRE infection (*n* = 388)	*P*
Age (mon), median (IQR)	7.6 (3.5–26.2)	7.4 (3.3–145.2)	0.032
Weight (kg), median (IQR)	7.0 (4.0–11.9)	7.1 (3.5–40.0)	0.035
Male, *n* (%)	32 (64.0)	229 (59.0)	0.499
Indications, *n* (%)			0.854
Biliary atresia	45 (90.0)	326 (84.0)	
Other chronic cholestatic diseases	1 (2.0)	18 (4.6)	
Inherited metabolic liver diseases	2 (4.0)	27 (7.0)	
Others	2 (4.0)	17 (4.4)	
Re-transplantation, *n* (%)	4 (8.0)	13 (3.4)	0.117
PELD scores, mean ± SD	13.7 ± 13.1	13.0 ± 10.6	0.678
Kasai before LT, *n* (%)	33 (66.0)	225 (58.0)	0.279
ICU stay before LT, *n* (%)	5 (10.0)	28 (7.2)	0.566
CRE rectal colonization, *n* (%)	16 (32.0)	29 (7.5)	< 0.001
Living donors, *n* (%)	33 (67.3)	271 (70.4)	0.661
ABOi, *n* (%)	9 (18.0)	70 (18.0)	0.994
Cold ischemia time (min)	80 (41–712)	78 (12–843)	0.665
RBC transfusion (U/kg)	0.43 (0.09–2.50)	0.37 (0–4.29)	0.007
Plasma transfusion (mL/kg)	42.2 (8.6–310.0)	31.7 (0–298.2)	0.052
Choledochojejunostomy, *n* (%)	50 (100.0)	368 (94.8)	0.149
Bile or intestinal leakage, *n* (%)	14 (28.0)	16 (4.1)	< 0.001
Respiratory RNA virus infection, *n* (%)	13 (26.0)	45 (11.6)	0.005

*PELD* pediatric end-stage liver disease, *LT* liver transplantation, *ICU* intensive care unit, *ABOi* ABO-incompatibility, *RBC* red blood cell, *RNA* ribonucleic acid, *CRE* carbapenem-resistant *Enterobacteriaceae*, *IQR* interquartile range, *SD* standard deviation

### Risk factors for CRE infections in pediatric liver transplant recipients

Univariate logistic regression analysis revealed potential risk factors. These included age, weight, CRE rectal colonization before liver transplantation, red blood cell transfusion, plasma transfusion, bile or intestinal leakage and respiratory RNA virus infection (*P* = 0.057, *P* = 0.041, *P* < 0.001, *P* = 0.021, *P* = 0.058, *P* < 0.001 and *P* = 0.006, respectively; Table 3). Furthermore, multivariate analysis identified CRE intestinal colonization before liver transplantation [odds ratio (OR) = 4.208, 95% confidence interval (CI) = 1.940–9.129; *P* < 0.001], bile or intestinal leakage (OR = 9.226; 95% CI = 3.774–22.556; *P* < 0.001) and respiratory RNA virus infection (OR = 2.205, 95% CI = 1.005–4.840; *P* = 0.049) as independent risk factors associated with post-transplant CRE infections. In addition, variance inflation factors (VIF) confirmed the absence of multicollinearity between clinically correlated variables: specifically, VIF = 1 for both the age-body weight pair and red blood cell-plasma transfusion pair.

**Table 3:** Univariate and multivariate logistic regression analyses of independent factors for CRE infections after liver transplantations

Variables	Univariate		Multivariate	
	OR (95% CI)	*P*	OR (95% CI)	*P*
Age	0.968 (0.935–1.001)	0.057	0.951 (0.886–1.021)	0.163
Weight	0.884 (0.786–0.995)	0.041	1.092 (0.836–1.426)	0.519
Male	1.234 (0.669–2.276)	0.500		
Biliary atresia	1.712 (0.653–4.484)	0.274		
Re-transplantation	2.598 (0.785–8.015)	0.121		
PELD scores	1.006 (0.979–1.033)	0.677		
Kasai before LT	1.406 (0.757–2.611)	0.280		
ICU stay before LT	1.429 (0.525–3.886)	0.485		
CRE intestinal colonization before LT	5.826 (2.880–11.783)	< 0.001	4.208 (1.940–9.129)	< 0.001
Living donors	0.868 (0.459–1.639)	0.662		
ABOi	0.997 (0.463–2.146)	0.994		
Cold ischemia time	1.000 (0.999–1.002)	0.664		
RBC transfusion	2.333 (1.133–4.802)	0.021	2.003 (0.856–4.687)	0.109
Plasma transfusion	1.006 (1.000–1.012)	0.058	1.000 (0.992–1.008)	0.963
Choledochojejunostomy	NA (0–NA)	0.998		
Bile or intestinal leakage before CRE infection	9.042 (4.084–20.016)	< 0.001	9.226 (3.774–22.556)	< 0.001
Respiratory RNA virus infection before CRE infection	2.678 (1.324–5.416)	0.006	2.205 (1.005–4.840)	0.049

*PELD* pediatric end-stage liver disease, *LT* liver transplantation, *ICU* intensive care unit, *ABOi* ABO-incompatibility, *RBC* red blood cell, *CRE* carbapenem-resistant *Enterobacteriaceae*, *RNA* ribonucleic acid, *OR* odds ratio, *CI* confidence interval

### Construction of CRE infection prediction models

**Fig. 2:**
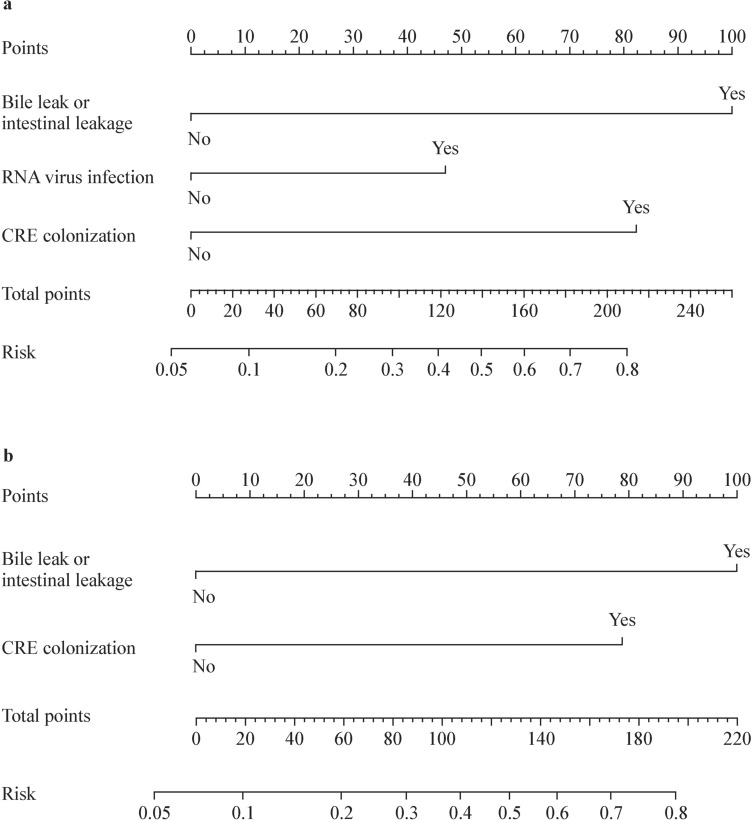
Nomograms for predicting CRE infections after liver transplantation in pediatric recipients across training and validation cohorts. **a** Model 1; **b** model 2. *CRE* Carbapenem-resistant *Enterobacteriaceae*, *RNA* respiratory ribonucleic acid

**Fig. 3:**
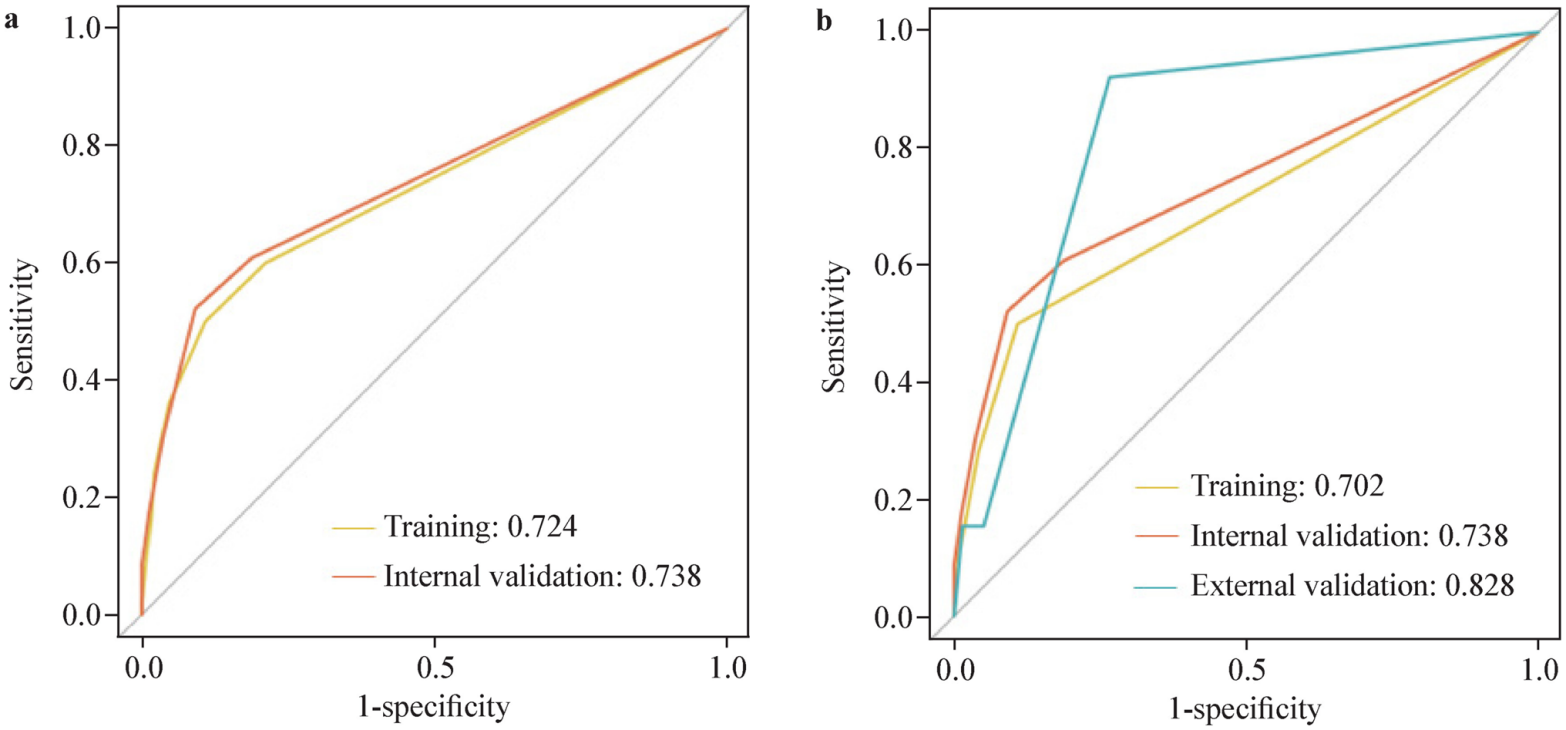
Receiver operating characteristic curves for Carbapenem-resistant *Enterobacteriaceae* infection predictive model 1 (**a**) and model 2 (**b**)

We constructed a nomogram (model 1) based on independent risk factors including CRE intestinal colonization before liver transplantation, bile or intestinal leakage and respiratory RNA virus infection to predict CRE infections in pediatric patients after liver transplantation (Fig. 2a). CRE intestinal colonization before liver transplantation and bile/intestinal leakage contributed most to the overall risk score in model 1. Therefore, a second nomogram (model 2) was constructed using only these two factors (Fig. 2b). AUCs of model 1 were 0.724 (95% CI = 0.646–0.802) and 0.738 (95% CI = 0.623–0.852) in the training and internal validation cohorts, respectively (Fig. 3a). Model 2 exhibited AUC values of 0.702 (95% CI = 0.628–0.775) and 0.738 (95% CI = 0.624–0.853) in the training and internal validation cohorts, respectively (Fig. 3b). Furthermore, the performance of model 2 was confirmed using an external validation cohort with an AUC of 0.828 (95% CI = 0.738–0.919, Fig. 3b). Model 1 demonstrated Brier scores of 0.086 and 0.088 in the training and internal validation cohorts, respectively. Model 2 showed Brier scores of 0.088, 0.088, and 0.071 in the training, internal validation, and external validation cohorts, respectively. Brier scores showed generally good alignment between the observed and predicted risks of CRE infection. In addition, we performed subgroup analyses based on operation time, using the median cutoff of 6 hours. In the external validation cohort, model 2 demonstrated superior performance for pediatric patients with prolonged operation time, achieving an AUC of 0.864 (95% CI = 0.840–0.888). In contrast, for shorter procedures, it showed reduced predictive accuracy with an AUC of 0.690 (95% CI = 0.658–0.722). Collectively this indicated that this model may perform better in pediatric patients with longer operative time.

### Postoperative complications and clinical outcomes in pediatric patients

After liver transplantation, there were no significant differences in acute rejection between patients with CRE infections and those not infected (4.0% vs. 6.2%, *P* = 0.755; Table 4). Patients who developed CRE infections often underwent longer periods of mechanical ventilation and post-transplant ICU stay (both *P* < 0.001). Seven patients with CRE infections died within 180 days of liver transplantation; eight patients not infected died over the same period (*P* < 0.001).

**Table 4:** Postoperative complications and clinical outcomes of patients with CRE and non-CRE infections in the training cohort

Outcomes	CRE infection (*n* = 50)	Non-CRE infection (*n* = 388)	*P*
Acute rejection within 180 d, *n* (%)	2 (4.0)	24 (6.2)	0.755
Mechanical ventilation (h), median (IQR)	16.5 (1.0–1584.0)	4.5 (0–1382.0)	< 0.001
Post-ICU stay (d), median (IQR)	7.0 (0–66.0)	5.4 (0–93.0)	< 0.001
Death within 180 d, *n* (%)	7 (14.0)	8 (2.1)	< 0.001

*CRE* carbapenem-resistant *Enterobacteriaceae*, *ICU* intensive care unit, *IQR* interquartile range

## Discussion

CRE infection poses a significant threat to patients undergoing liver transplantation [19]. The present large-scale study identified CRE intestinal colonization before liver transplantation, post-transplant bile leak or intestinal leakage and respiratory RNA virus infection as independent risk factors of CRE infection in pediatric liver transplant recipients. Moreover, this study constructed prediction models based on these risk factors. These models performed well in training and internal validation cohorts. In addition, the model that included only CRE intestinal colonization and bile or intestinal leakage also achieved satisfactory performance in the internal cohorts; its prediction efficacy was well validated using an external cohort.

As demonstrated in previous studies, CRE intestinal colonization before liver transplantation is significantly associated with CRE bloodstream infection after liver transplantation [5, 20, 21]. Preoperative CRE colonization in pediatric liver transplantation necessitates active pre-surgical detection as these recipients may benefit from preemptive therapy [22].

As living donor and choledochojejunostomy procedures are frequently performed in pediatric liver transplantation, there is an increased risk of post-transplant bile or intestinal leakage [23, 24]. These complications can significantly increase the risk of CRE infections. Moreover, a previous study revealed that increased exposure to antibiotics and biliary interventions may further increase the risk of CRE infections [25]. Protection of bile ducts in liver transplantation is a critical process [26] but where this fails complications require timely and effective intervention [27].

Immunosuppressed pediatric recipients are more susceptible to respiratory RNA virus infections; this was included in our analysis [28]. Severe respiratory RNA virus infections increase the risk of CRE infections [29]. Prevention of RNA virus infections and administering targeted antibiotics in the event of such infections is paramount. In addition, the safety and efficacy of drugs targeting these infections requires careful consideration in pediatric recipients concurrently using immunosuppressants [30].

It is noteworthy that although the current management of CRE infections has developed in recent years the mortality rate, which amounted to 14% in the present study, remains high. Prolonged postoperative ICU stay and ventilation time represent a concerning economic burden for those affected. Poor outcomes underscore the importance of focusing on CRE infections. To bridge the gap between our predictive model and clinical implementation, we plan to conduct prospective clinical studies to validate several interventions. For pediatric patients at high-risk, we recommend: (1) immediate isolation, including single-room isolation with dedicated medical equipment and staff, to prevent potential transmission; (2) enhanced screening protocol including regular rectal swabs and environmental cultures of high-touch surfaces like bedrails and monitors; (3) preemptive therapy as a potential strategy to prevent progression from early colonization to invasive infections, particularly when initiated promptly upon detection of early signs of infection. We plan to conduct a multicenter randomized controlled trial to evaluate the efficacy of preemptive therapy, initiated when early signs of infection are present, compared with standard monitoring in high-risk patient populations. This investigation aims to transform current CRE management from passive to active through screening of high-risk populations and monitoring of both microbial and host factors. Data from this study may also establish novel infection prevention strategies for immunocompromised patients.

There are several limitations to the present study. First, our retrospective findings require prospective validation. Second, respiratory RNA virus detection may not be consistently implemented in some centers; this hinders accurate retrospective validation of the importance of these viruses. Future studies on the role of respiratory RNA viruses are needed. Third, optimal prevention strategies for pediatric patients at a high risk of CRE infection remains unclear; this requires further research.

In conclusion, this study identified risk factors and constructed prediction models for CRE infections occurring after liver transplantation in pediatric populations. These findings could aid identification of pediatric recipients at a higher risk of developing CRE infections and further aid uptake of protective measures, ultimately contributing to improved quality of life after liver transplantation.

## Data Availability

The datasets used during the current study are available from the corresponding author on reasonable request.

## References

[1] Pouch SM, Satlin MJ. Carbapenem-resistant *Enterobacteriaceae* in special populations: solid organ transplant recipients, stem cell transplant recipients, and patients with hematologic malignancies. Virulence. 2017;8:391–402. 27470662 10.1080/21505594.2016.1213472 PMC5477691

[2] Huemer M, Mairpady Shambat S, Brugger SD, Zinkernagel AS. Antibiotic resistance and persistence-Implications for human health and treatment perspectives. EMBO Rep. 2020;21:e51034. 33400359 10.15252/embr.202051034 PMC7726816

[3] Aguado JM, Silva JT, Fernandez-Ruiz M, Cordero E, Fortun J, Gudiol C, et al. Management of multidrug resistant Gram-negative bacilli infections in solid organ transplant recipients: SET/GESITRA-SEIMC/REIPI recommendations. Transplant Rev (Orlando). 2018;32:36–57. 28811074 10.1016/j.trre.2017.07.001

[4] Anesi JA, Lautenbach E, Thom KA, Tamma PD, Blumberg EA, Alby K, et al. Clinical outcomes and risk factors for carbapenem-resistant *Enterobacterales* bloodstream infection in solid organ transplant recipients. Transplantation. 2023;107:254–63. 35856636 10.1097/TP.0000000000004265 PMC9772065

[5] Giannella M, Freire M, Rinaldi M, Abdala E, Rubin A, Mularoni A, et al. Development of a risk prediction model for carbapenem-resistant *Enterobacteriaceae* infection after liver transplantation: a multinational cohort study. Clin Infect Dis. 2021;73:e955–66. 33564840 10.1093/cid/ciab109

[6] Chen Y, Wang WL, Zhang W, Zhang YT, Tang SX, Wu PP, et al. Risk factors and outcomes of carbapenem-resistant *Enterobacteriaceae* infection after liver transplantation: a retrospective study in a Chinese population. Infect Drug Resist. 2020;13:4039–45. 33204121 10.2147/IDR.S278084 PMC7666982

[7] Giannella M, Bartoletti M, Campoli C, Rinaldi M, Coladonato S, Pascale R, et al. The impact of carbapenemase-producing *Enterobacteriaceae* colonization on infection risk after liver transplantation: a prospective observational cohort study. Clin Microbiol Infect. 2019;25:1525–31. 31039445 10.1016/j.cmi.2019.04.014

[8] Centers for Disease Control and Prevention (CDC). Guidance for control of infections with carbapenem-resistant or carbapenemase-producing *Enterobacteriaceae* in acute care facilities. MMWR Morb Mortal Wkly Rep. 2009;58:256–60. 19300408

[9] Taylor SA, Venkat V, Arnon R, Gopalareddy VV, Rosenthal P, Erinjeri J, et al. Improved outcomes for liver transplantation in patients with biliary atresia since pediatric end-stage liver disease implementation: analysis of the society of pediatric liver transplantation registry. J Pediatr. 2020;219:89–97. 32005543 10.1016/j.jpeds.2019.12.023

[10] Ronan V, Yeasin R, Claud EC. Childhood development and the microbiome-the intestinal microbiota in maintenance of health and development of disease during childhood development. Gastroenterology. 2021;160:495–506. 33307032 10.1053/j.gastro.2020.08.065 PMC8714606

[11] Fu P, Xu H, Jing C, Deng J, Wang H, Hua C, et al. Bacterial epidemiology and antimicrobial resistance profiles in children reported by the ISPED program in China, 2016 to 2020. Microbiol Spectr. 2021;9:e0028321. 34730410 10.1128/Spectrum.00283-21 PMC8567242

[12] Tanimura S, Chuah SL, Shimizu S, Sakamoto S, Kasahara M, Nakagawa S. Pattern of infection after pediatric liver transplant and its associated risk factors. Pediatr Transplant. 2025;29:e70032. 39837776 10.1111/petr.70032

[13] Pouch SM, Patel G, AST Infectious Diseases Community of Practice. Multidrug-resistant Gram-negative bacterial infections in solid organ transplant recipients-guidelines from the American Society of Transplantation Infectious Diseases Community of Practice. Clin Transplant. 2019;33:e13594. 31102483 10.1111/ctr.13594

[14] Humphries R, Bobenchik AM, Hindler JA, Schuetz AN. Overview of changes to the clinical and laboratory standards institute performance standards for antimicrobial susceptibility testing, M100, 31st edition. J Clin Microbiol. 2021;59:e0021321. 34550809 10.1128/JCM.00213-21 PMC8601225

[15] Huang J, Wang H, Fan ST, Zhao B, Zhang Z, Hao L, et al. The national program for deceased organ donation in China. Transplantation. 2013;96:5–9. 23743728 10.1097/TP.0b013e3182985491

[16] Yuan X, Xu Q, Du F, Gao X, Guo J, Zhang J, et al. Development and validation of a model to predict cognitive impairment in traumatic brain injury patients: a prospective observational study. EClinicalMedicine. 2025;80:103023. 39850016 10.1016/j.eclinm.2024.103023 PMC11753911

[17] Singh SK, Poddar U, Mishra R, Srivastava A, Yachha SK. Ascitic fluid infection in children with liver disease: time to change empirical antibiotic policy. Hepatol Int. 2020;14:138–44. 31290071 10.1007/s12072-019-09968-x

[18] Zhang W, Chen Y, Zhang Y, Wang R, Wang W, Bai X, et al. Carbapenems versus cephalosporin or piperacillin-tazobactam as perioperative antibiotic prophylaxis in liver transplant recipients with model for end-stage liver disease scores of ≥30: a retrospective study in a Chinese population. Infect Drug Resist. 2022;15:4487–94. 35983301 10.2147/IDR.S373773 PMC9380821

[19] Men TY, Wang JN, Li H, Gu Y, Xing TH, Peng ZH, et al. Prevalence of multidrug-resistant gram-negative bacilli producing extended-spectrum beta-lactamases (ESBLs) and *ESBL* genes in solid organ transplant recipients. Transpl Infect Dis. 2013;15:14–21. 23013385 10.1111/tid.12001

[20] Liu J, Zhang H, Feng D, Wang J, Wang M, Shen B, et al. Development of a risk prediction model of subsequent bloodstream infection after carbapenem-resistant *Enterobacteriaceae* isolated from perianal swabs in hematological patients. Infect Drug Resist. 2023;16:1297–312. 36910516 10.2147/IDR.S400939 PMC9999719

[21] Sun Y, Yu L, Gao W, Cai J, Jiang W, Lu W, et al. Investigation and analysis of the colonization and prevalence of carbapenem-resistant *Enterobacteriaceae* in pediatric liver transplant recipients. Infect Drug Resist. 2021;14:1957–66. 34079305 10.2147/IDR.S304998 PMC8164869

[22] Yang TT, Luo XP, Yang Q, Chen HC, Luo Y, Zhao YM, et al. Different screening frequencies of carbapenem-resistant *Enterobacteriaceae* in patients undergoing hematopoietic stem cell transplantation: which one is better? Antimicrob Resist Infect Control. 2020;9:49. 32183898 10.1186/s13756-020-0706-0 PMC7077122

[23] Feier FH, Chapchap P, Pugliese R, Da Fonseca EA, Carnevale FC, Moreira AM, et al. Diagnosis and management of biliary complications in pediatric living donor liver transplant recipients. Liver Transpl. 2014;20:882–92. 24760734 10.1002/lt.23896

[24] Guirguis RN, Nashaat EH, Yassin AE, Ibrahim WA, Saleh SA, Bahaa M, et al. Biliary complications in recipients of living donor liver transplantation: a single-centre study. World J Hepatol. 2021;13:2081–103. 35070010 10.4254/wjh.v13.i12.2081 PMC8727210

[25] Pereira MR, Scully BF, Pouch SM, Uhlemann AC, Goudie S, Emond JE, et al. Risk factors and outcomes of carbapenem-resistant *Klebsiella pneumoniae* infections in liver transplant recipients. Liver Transpl. 2015;21:1511–9. 26136397 10.1002/lt.24207 PMC4896355

[26] Op Den Dries S, Sutton ME, Lisman T, Porte RJ. Protection of bile ducts in liver transplantation: looking beyond ischemia. Transplantation. 2011;92:373–9. 10.1097/TP.0b013e318223a384 21629175

[27] Neto JS, Costa CM, De Assis AM, Pugliese R, Benavides MR, Carnevale FC, et al. Treatment strategies for bile leak following pediatric liver transplantation. Pediatr Transplant. 2024;28:e14814. 38895799 10.1111/petr.14814

[28] Danziger-Isakov L, Steinbach WJ, Paulsen G, Munoz FM, Sweet LR, Green M, et al. A multicenter consortium to define the epidemiology and outcomes of pediatric solid organ transplant recipients with inpatient respiratory virus infection. J Pediatr Infect Dis Soc. 2019;8:197–204. 10.1093/jpids/piy024 PMC7107524 29538674

[29] Lai CC, Wang CY, Hsueh PR. Co-infections among patients with COVID-19: The need for combination therapy with non-anti-SARS-CoV-2 agents? J Microbiol Immunol Infect. 2020;53:505–12. 32482366 10.1016/j.jmii.2020.05.013 PMC7245213

[30] Lombardi A, Alagna L, Palomba E, Viero G, Tonizzo A, Mangioni D, et al. New antibiotics against multidrug-resistant Gram-negative bacteria in liver transplantation: clinical perspectives, toxicity, and PK/PD properties. Transpl Int. 2024;37:11692. 38362283 10.3389/ti.2024.11692 PMC10867129

